# Short-Wavelength Light–Blocking Filters and Oral Melatonin Administration in Patients With Retinitis Pigmentosa: Protocol for a Randomized Controlled Trial

**DOI:** 10.2196/49196

**Published:** 2023-11-16

**Authors:** Salvador Pastor-Idoate, Milagros Mateos-Olivares, Eva María Sobas, Miguel Marcos, Alfredo Toribio, José Carlos Pastor, Ricardo Usategui Martín

**Affiliations:** 1 Institute of Applied Ophthalmobiology University of Valladolid Valladolid Spain; 2 Department of Ophthalmology Clinical University Hospital of Valladolid Valladolid Spain; 3 Networks of Cooperative Research oriented to Health Results National Institute of Health Carlos III Madrid Spain; 4 European Reference Network dedicated to Rare Eye Diseases Valladolid Spain; 5 Department of Ophthalmology Clinical University Hospital of Caceres Caceres Spain; 6 Nursing School University of Valladolid Valladolid Spain; 7 Department of Internal Medicine University Hospital of Salamanca Salamanca Spain; 8 Institute of Biomedical Research of Salamanca University of Salamanca Salamanca Spain; 9 Federation of Associations of Hereditary Retinal Dystrophies in Spain Valladolid Spain; 10 Department of Cellular Biology Faculty of Medicine University of Valladolid Valladolid Spain

**Keywords:** retinitis pigmentosa, oral melatonin, short-wavelength light, intrinsically photosensitive retinal ganglion cells, ipGC, retinal dystrophy, quality of life, rare eye disease, eye disease, burden, health care system, intervention, treatment, vision loss, psychological stress, sleep disorder, eye, retina, cornea, lens, glaucoma, cataract

## Abstract

**Background:**

The medical community is beginning to recognize that retinitis pigmentosa (RP), due to its disabling progression, eventually leads to a reduction in the patient´s quality of life, a direct economic impact, and an increase in the burden on the health care system. There is no curative treatment for the origin of the disease, and most of the current interventions fail in reducing the associated negative psychological states, such as anxiety and depression, which lead to increased variability of vision and pose a continuous threat to the patient’s independence.

**Objective:**

The aim of this study is to assess the effect of oral melatonin (OM) administration alone and combined with short-wavelength light (SWL)–blocking filters on patients with RP and test their effectiveness in improving the level of stress and sleep problems in many of these patients.

**Methods:**

We have developed a low-cost therapy protocol for patients with RP with sleep disorders and negative psychological stress. Patients will be randomized to receive a combined intervention with SWL-blocking filters and OM, SWL-blocking filters alone, or OM alone. There will also be a nonintervention arm as a control group. This study will be conducted across 2 retinal units in patients with RP with sleep disorders and high perceived stress and anxiety score reports. Patients will be assessed in the preintervention period, weekly during the 4 weeks of intervention, and then at 6 months postintervention. The primary outcomes are the differences in changes from baseline to postintervention in hormone release (α-amylase, cortisol, and melatonin) and sleep quality, as measured with the visual analog scale. Secondary outcome measures include clinical macular changes, as measured with optical coherence tomography and optical coherence tomography angiography; retinal function, as measured using the visual field and best-corrected visual acuity; sleep data collected from personal wearables; and several patient-reported variables, such as self-recorded sleep diaries, quality of life, perceived stress, and functional status.

**Results:**

This project is still a study protocol and has not yet started. Bibliographic research for information for its justification began in 2020, and this working group is currently seeking start-up funding. As soon as we have the necessary means, we will proceed with the registration and organization prior to the preliminary phase.

**Conclusions:**

In this feasibility randomized clinical controlled trial, we will compare the effects of SWL blocking alone, administration of OM alone, and a combined intervention with both in patients with RP. We present this study so that it may be replicated and incorporated into future studies at other institutions, as well as applied to additional inherited retinal dystrophies. The goal of presenting this protocol is to aid recent efforts in reducing the impact of sleeping disorders and other psychological disorders on the quality of life in patients with RP and recovering their self-autonomy. In addition, the results of this study will represent a significant step toward developing a novel low-cost therapy for patients with RP and validating a novel therapeutic target.

**International Registered Report Identifier (IRRID):**

PRR1-10.2196/49196

## Introduction

### Background and Rationale

Retinitis pigmentosa (RP) is the most frequent form of inherited retinal dystrophy (IRD) and the main cause of hereditary blindness, with more than 1.5 million patients affected worldwide and a prevalence of 1:4000 people [1]. The term was coined by Donders in 1857 [2] and compounds a group of heterogeneous diseases characterized by a progressive degeneration of photoreceptors, starting with rods and eventually affecting cones as well. This is the mechanism responsible for the progressive loss of vision function [3].

RP can have bilateral or, less frequently, unilateral involvement and can occur isolated or as part of a syndrome [1,4,5]. It is caused by more than 3000 mutations in over 70 genes, creating a wide heterogeneity among patients with RP [6].

RP affects mainly the outer retina, but in the final stages of the disease, atrophy and damage ultimately affect the inner retina, including retinal ganglion cells [7]. Intrinsically photosensitive retinal ganglion cells (ipGCs) constitute a special type of photoreceptors within the retina that contain the melanopsin photopigment [8,9]. Although they represent less than 10% of the total ganglion cells, they play a key role in regulating the pupillary reflex, circadian photoentrainment, mood, and diverse cognitive functions [9-16]. Alterations in circadian rhythms, partially dependent on ipGC integrity, have been previously reported in different ocular diseases and blindness [17-22]. These patients usually do not properly receive light signals that ordinarily synchronize their internal circadian clock to the 24-hour day, causing sleep-wake cycle abnormalities and recurring blocks of days with severe insomnia and excessive daytime sleepiness [21]. This circadian clock impairment could ultimately affect the quality of life due to sleep disorders and could also involve cardiovascular problems or emotional disorders or trigger or accelerate the pathology progression in neurodegenerative diseases [7,23,24].

The few therapeutic options for patients with RP currently available in daily clinical practice just retard disease progression and are limited. There is no therapy nowadays that restores vision, so the prognosis is poor [3]. Because of the chronic nature of this disease and its disabling progression, there has been great interest in the development of new strategies to help patients cope with the social and psychological impacts of blindness.

Among nonpharmacological strategies aimed at slowing down the degenerating process are those that are based on light protection. Some pieces of evidence indicate that some hereditary pigmentary retinopathies are partly light dependent [25,26]. Violet (400-440 nm) and blue (440-500 nm) light constitute the lower wavelength of the visible spectrum, which carries the highest amount of energy per photon [27]. The possible role of short-wavelength light (SWL) in the photochemical damage to retinal tissue and retinal pigment epithelium (RPE) cells has been studied, with high-energy SWL (415-455 nm) being the most harmful to the eye (the second type of phototoxicity or blue-light hazard) [28-30]. Blue-violet light could cause oxygen-dependent retinal injury, acting on specific chromophores, and *N*-retinylidene-*N*-retinylethanolamine (A2E) is considered the most important target molecule. Reactive oxygen species (ROS) initiate the activity of cysteine-dependent proteases, ultimately leading to apoptosis and cell death [28,31-34]. Mitochondria from photoreceptors, retinal ganglion cells, and RPE cells are the main targets of blue light–associated oxygen free radicals [35-37]. Moreover, photophobia, a common unpleasant symptom in patients with RP, has been described to be caused primarily by SWL in its interaction with S-cones from the retina [38]. In addition, in experimental RP models, limiting SWL exposure by the application of optical filters has been demonstrated to slow retinal degeneration [39].

However, some studies have shown that longer blue wavelengths of the visible spectrum (465-495 nm) are essential for the circadian rhythm, pupillary reaction, color discrimination, and night vision [7,23,24,40-43]. Numerous studies have demonstrated their close association to circadian rhythm regulation, alertness, memory, and cognition [44,45]. Therefore, blue-light exposure could regulate the secretion of melatonin in the pineal gland through the retinal–suprachiasmatic nucleus (SCN)–pineal axis, modulating cortisol expression through the hypothalamic–pituitary–adrenal (HPA) axis or the autonomous nervous system [7,23,24,44]. These responses are driven primarily by intrinsically photosensitive retinal ganglion cells (ipGCs), which are more sensitive to 480 nm SWL and remain functional in late stages of the disease [7]. In contrast, melatonin suppression has a peak sensitivity of roughly 460 nm in humans, the same spectrum that has been shown to reset the circadian pacemaker and directly enhance alertness [46].

In consequence, evening SWL exposure in daily life may affect sleep, hormone release, and the circadian rhythm in patients with RP. In fact, it has been reported that wearing SWL-blocking glasses that virtually eliminate SWL is effective in improving sleep, the circadian rhythm, and mood. Three randomized controlled trials (RCTs) have demonstrated that SWL-blocking glasses bring about significant improvement in sleep quality in participants with insomnia [42,43,47].

Among pharmacological interventions, the trophic and antioxidant effects of vitamins or nutritional supplements have been evaluated in patients with RP to demonstrate a protective action on cone death [48-50]. Accumulating evidence indicates that melatonin therapy is a promising approach in the treatment of various ocular degenerative disorders (eg, age-related macular degeneration, diabetic retinopathy, and RP) since it can exert a protective effect against damage to RPE cells evoked by ROS, but it has also been reported to increase ROS-induced damage to photoreceptors and RPE cells [51-55].

Melatonin is an endogenous hormone that has been implicated in a variety of biological processes, including antioxidant, anti-inflammatory, and neuroprotective activities [56,57]. Melatonin is released from the pineal gland in a circadian fashion, which is controlled by the SCN. However, many other tissues and organs also synthesize melatonin. In the retina, it is mainly synthesized by photoreceptors and is involved in the modulation of neuronal activities. Its circadian production results in a high concentration at night and a low level during the day, although its amount is small compared to its pineal counterpart [7,51,58].

Oxidative damage has been proposed to be an important contributor to cone death in RP, and antioxidants may prevent cone damage in RP by reacting with free radicals produced during light absorption [59]. Melatonin behaves like synthetic mitochondria-targeted antioxidants, which are concentrated in the mitochondria at relatively high levels [51,58]. It exerts its antioxidant actions in the eye via several mechanisms, including direct ROS scavenging, stimulating antioxidant enzymes, improving the functioning of the mitochondrial electron transport chain (ETC), reducing the extent of electron leakage from the mitochondrial complexes, and improving the efficacy of other antioxidants. As mentioned before, mitochondria from photoreceptors, retinal ganglion cells, and RPE cells are the main targets of ROS produced by the ETC, and thus, melatonin may prevent mitochondrial damage in patients with RP [7,51,58,60,61].

However, it has also been demonstrated that daily administration of appropriately timed melatonin can synchronize the circadian clock [62-66] in patients with sleep disorders; its use is even recommended by the Standards of Practice Committee of the American Academy of Sleep Medicine [67]. The phase-shifting effects of melatonin are essentially opposite to those of light. It can phase-shift the endogenous circadian clock and, in the absence of light, entrain the sleep-wake and neuroendocrine rhythms (cortisol) [61]. Therefore, it is likely that OM may also be effective in sleep and circadian rhythm management in patients with RP. However, to the best of our knowledge, no previous RCT has investigated the potential additive benefits of adjunctive SWL-blocking glasses plus OM in improving sleep, perceived stress, and the circadian rhythm in patients with RP.

The aim of this study is to assess the efficacy of OM combined with SWL-blocking filters in patients with RP who have sleep disorders and stress compared to any intervention alone or no intervention. We hypothesized that both interventions would improve their sleep quality, reduce their perceived stress levels, and shift their circadian rhythm to morningness.

### Need for a Trial (Innovation Aspect)

RP is the commonest of inherited retinal disorders, with a great economic impact and poor associated quality of life [68]. In addition, there is a lack of standard treatment with a good level of efficacy data, which brings a fruitful opportunity to add new knowledge and pursue evidence-based interventions.

The innovation in this study is based on a noninvasive approach—SWL-blocking filters combined with OM. This treatment protocol might represent a better option for patients with RP, especially those with moderate or severe RP, who present with greater functional disability, glare symptoms, and variations in cortisol, salivary α-amylase (sAA), and melatonin levels.

Although a few trials have assessed the efficacy of different vitamins and melatonin in visual and sleep pattern anomalies in patients diagnosed with RP, none of them used a similar combined therapy and even fewer focused on variations in cortisol, sAA, and melatonin levels or determined the impact of these interventions on psychological factors. This study aims to fill this gap in the literature by enhancing the level of evidence with a double-blind RCT that evaluates the benefits that OM combined with SWL-blocking filters may bring to patients with moderate or severe RP.

### Significance/Impact of the Study

RP is accompanied by significant disability, greater dependency, and a decrease in the health-related quality of life, with increased rates of admission to institutional facilities [68-70]. It is also considered a public health issue and a primary cause of headaches, sleep disturbances, and increased rates of depression [71]. Patients with RP also face difficulties in performing daily activities and increased risk of cardiovascular, metabolic, and psychiatric disorders [68-70]. Productivity losses for patients with visual impairment are likely to be amplified in patients with RP because of the early age at onset, vision challenges at school, and loss of visual function without some type of rehabilitation during prime working years [68-70].

The annual cost of adult vision problems for the National Health Service (NHS) is around US $51.4 billion. Among adults with retinal diseases, the annual total health care costs were estimated to be US $7317 higher per patient with RP, increasing the community costs by around 2.5 times because of the number of years of blindness experienced [72].

SWL-blocking filters and OM have been playing an important role in the treatment of several kinds of retinal conditions and sleep disabilities [42,43,47,60,61,73]. Unlike other expensive treatments for sleep disorders, anxiety, and depression, SWL-blocking filters or OM may provide effective control of the human circadian clock through changes in the HPA axis or the retinal–SCN–pineal axis, which could be positively correlated with improvement in the quality of life and visual function disability.

Some studies suggest a multidisciplinary treatment program that includes specialists from different areas of expertise [74]. This program provides a combination of drug treatment and SWL-blocking filters with an adequate assessment of the grade of severity (via clinical assessment) and biochemical stress (by analyzing biomarkers) and determines the impact of psychological factors on patients with RP. It could be considered a good alternative for patients with moderate and severe RP who have not benefited from expensive therapies (eg, bionic eye or genetic therapy) individually. Therefore, in this study, we suggest mimicking a multidisciplinary treatment by combining treatments and analyzing the impact of biochemical and psychological factors on anxiety and sleep disability.

### Choice of Comparators

To date, therapeutic approaches for RP are restricted to slowing down the degenerative process; treating ocular complications, such as cataracts and macular edema; and helping patients cope with the social and psychological impacts of blindness. In addition, the evidence from RCTs on the visual protective effects of vitamins and nutritional supplementations in patients with RP is limited and in some cases contradictory. Moreover, as mentioned before, few studies on the effects of blocking SWL on visual discrimination capacity in patients with RP have been reported. In addition, in clinical practice, there is insufficient experience to recommend the use of optical absorbing filters, since their potential effects on regulating the human internal circadian (24-hour) clock in patients with RP are yet unknown.

For this reason, the intervention groups will consist of SWL blocking alone (SWL group), OM therapy alone (OM group), and SWL blocking plus OM therapy (SWL+OM group) in order to determine whether there is any summative effect. No intervention has been chosen for comparison to avoid performance bias and other biases related to loss of blinding in the trial. Therefore, as several treatments have been proposed to slow down the degenerative process and the current evidence from their results is still limited, no intervention as the control group is justified for this study.

## Methods

### Overview

This parallel-group RCT will be performed at a single institution, which is a tertiary referral hospital with an affiliated research institute (Institute of Applied Ophthalmobiology [IOBA], University of Valladolid). The retinal units in both institutions are highly specialized to manage inherited retinal dystrophies and other related retinal eye diseases. Additionally, the IOBA has a clinical trial unit that could help with the local ethical requirements for running a clinical trial and any oversight or external auditing of data that is needed.

**Figure 1:**
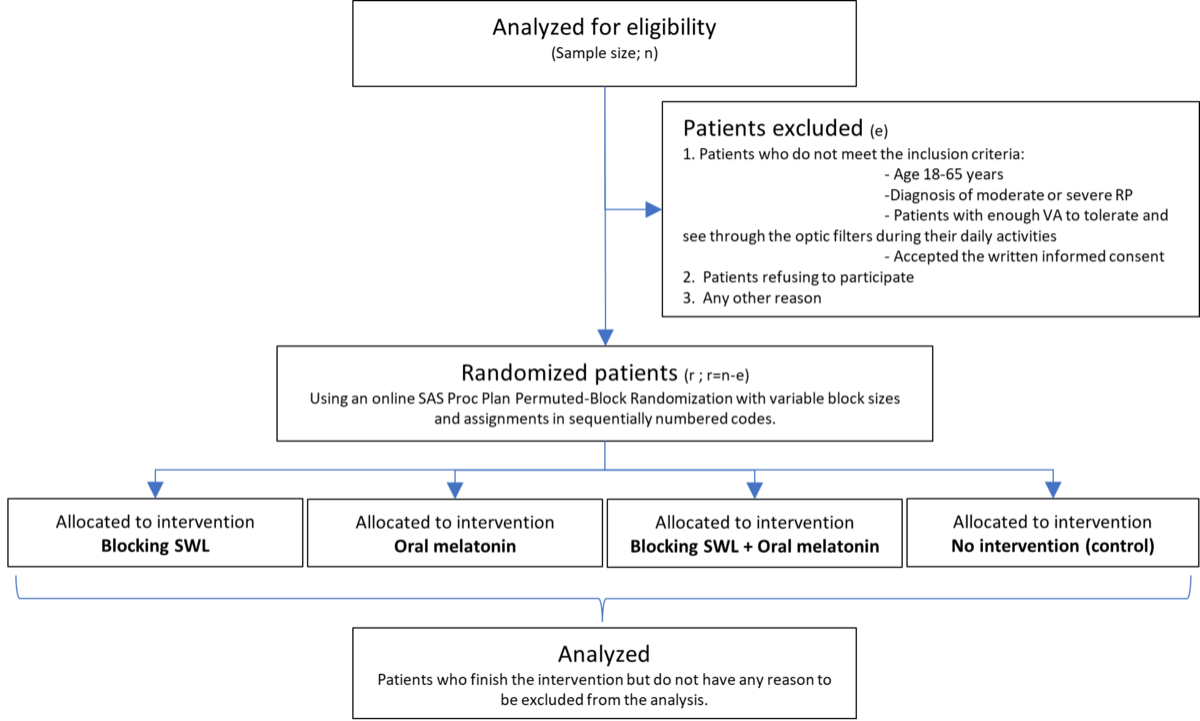
Flow diagram illustrating the study protocol. At the allocation to interventions, the number of patients who receive the intervention will be registered, as well as those who do not receive it (together with the reason it was not applied) and those who get lost in the follow-up or discontinue the intervention. In addition, unbearable side effects and SUSARs would be appropriately notified and registered. e: patients excluded; r: randomized patients; RP: retinitis pigmentosa; SUSAR: suspected unexpected serious adverse reaction; SWL: short-wavelength light; VA: visual acuity.

There will be 4 parallel groups of patients. Recruitment will be facilitated by a local foundation for patients with retinitis pigmentosa called the Retina Castilla y León (ReCYL) and will be tightly supervised by an ophthalmologist with specialization in retinal degenerations. Figure 1 provides a summary of the clinical pathways.

A draft intervention protocol will be developed for each group, covering preintervention, intervention, postintervention, and discharge settings based on the literature. A steering committee comprising ophthalmologists, opticians, nurses, psychologists, and the research team will be created to develop these protocols. Additionally, 2 ophthalmology residents will be included. Finally, 1 patient with RP, who is the current president of the Federation of Inherited Retinal Dystrophies’ Associations, will be included in the steering committee. The steering committee members are experts in their respective fields and have used the existing literature (summarized in the *Background and Rationale* section) to inform the inclusion of the different interventions in the protocols. The draft intervention protocols are presented in Table 1. Although the choice of each intervention in these protocols will be informed by the literature [7,28,30,36,51,57,67,75-82] and expert consensus of the steering committee, the study will assess the efficacy of combined interventions in sleep disorders and stress control in a large group of patients with RP. Before the intervention period, all patients will be asked to complete sleep diaries for 2 weeks. In addition, patients in the intervention groups (SWL, OM, SWL+OM) must complete them during the intervention period (30 days). Patients from the SWL and SWL+OM groups will wear SWL-restricting goggles in bright-light conditions and when using light-emitting diode (LED) electronic devices for the 30 days of intervention. Patients from the OM and SWL+OM groups will take 3 pills of Circadin 2 mg (OM) every evening at 10:00 P.M. with a margin of 30 minutes before and after.

**Figure 2:**
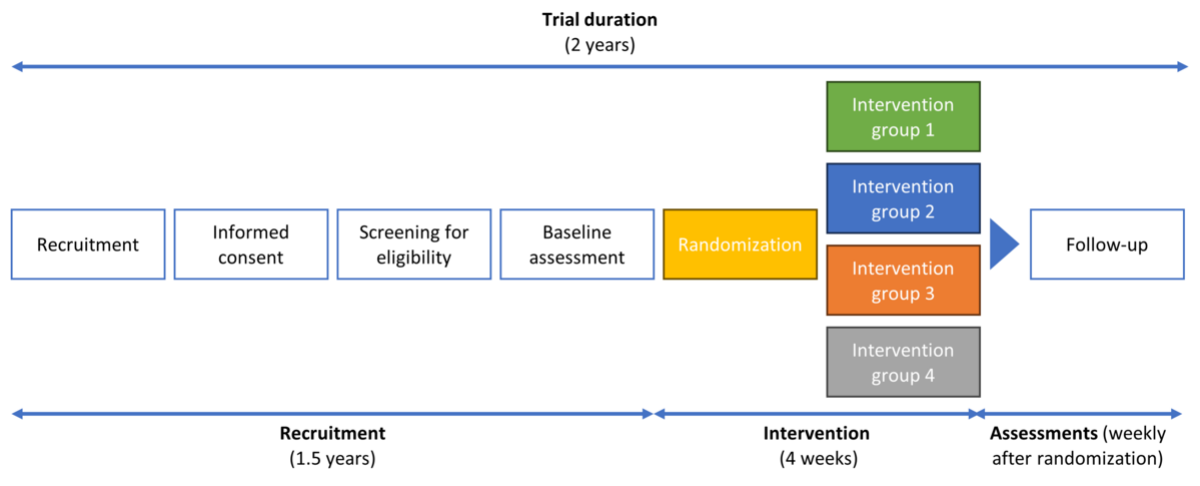
RCT timeline. RCT: randomized controlled trial.

A preintervention time of 1.5 years will be necessary for recruitment, informed consent, eligibility screening, and baseline data collection. Next, patients will be randomized to 4 groups in a 1:1 ratio. The treatment groups will include SWL with or without the use of OM. The control group will include patients who are eligible for the study but not willing to be randomized to an intervention sleep and stress management pathway. When the intervention phase (4 weeks) is over, all patients will be followed up for 6 months via standard monitoring. Figure 2 provides a summary of the RCT timeline.

**Table 1:** Protocols of the intervention.

Group	Preintervention period (2 weeks)	Intervention period (30 days)
SWL^a^: optical SWL filters	Sleep diary	Sleep diary, goggles
OM^b^	Sleep diary	Sleep diary, OM
SWL+OM: optical SWL filters and OM	Sleep diary	Sleep diary, goggles, OM
Control: no intervention	Sleep diary	None

^a^SWL: short-wavelength light.

^b^OM: oral melatonin.

### Study Population

The study population will comprise patients aged 18-65 years with a history of moderate or severe RP with a grading severity scale score of >1 point according to Iftikhar et al’s [83] scoring criteria. Eligible patients who provide written informed consent will be randomized to 1 of the 4 study groups. Outcomes will be assessed at multiple time points: at baseline in the preintervention period; then at 1, 2, 3, and 4 weeks of the intervention period; and then at 6 months postintervention.

### Patient Selection, Recruitment, Randomization, and Blinding

#### Eligibility

Eligibility will be determined based on the inclusion and exclusion criteria listed in Table 2. Eligible participants will be identified after initial evaluation by a staff ophthalmologist, determined to satisfy 1 of the qualifying procedures, and approached for informed consent by the ophthalmologist and research staff. At this time, patients will watch an introductory video explaining the purpose of the clinical trial. The physician also will lead an in-depth preintervention discussion explaining each approach to stress and sleep disability (SWL and OM vs control) and postintervention expectations. If patients decline to participate in the RCT, they will be presented with the option of enrolling in the control group (no intervention). There will be no restrictions on patient care due to enrollment in the study.

**Table 2:** Inclusion and exclusion criteria at screening.

Inclusion criteria	Exclusion criteria
Age 18-65 years, from ReCYL^a^ Diagnosis of moderate or severe RP^b^ (grading severity scale score>1 point) Visual function enough to tolerate and see through the optic filters during daily activities Written informed consent provided	Underwent any ocular surgery within the previous 6 months Involvement in any other ongoing clinical trial Pregnancy and lactation or under hormonal treatment Allergy or hypersensitivity to melatonin Previous or current therapy with melatonin Autoimmune diseases NSAID^c^ and others anti-inflammatory drug treatment Psychiatric disorders or under any psychotropic medication

^a^ReCYL: Retina Castilla y León.

^b^RP: retinitis pigmentosa.

^c^NSAID: nonsteroidal anti-inflammatory drug.

#### Recruitment

##### Screening Visit

Patients will undergo an initial screening visit that includes a semistructured interview, a series of questionnaires, a complete ophthalmic examination, and laboratory assessment. Eligible patients will be provided with a verbal description of the trial and presented with a comprehensible informed consent form.

##### Semistructured Interview

All patients will undergo a semistructured interview to collect medical (signs and symptoms, personal and family records, coexisting pathology, previous or current medication, etc), demographic (age, gender, ethnicity, etc), socioeconomic (relationship status, educational attainment, employment status, etc), and lifestyle (physical exercise, consumption of drugs, use of hair dyes, BMI, etc) data.

##### Ophthalmic Examination

All patients will undergo a complete ophthalmological examination, which will include the best-corrected visual acuity (BCVA) with the Early Treatment of Diabetic Retinopathy Study (ETDRS) letter score, a slit lamp examination, intraocular pressure measurement, fundoscopy, contrast sensitivity with a CSV-1000 chart (Vectorvision), and a glare test with the MonPack 3 device (Metrovision).

##### Ancillary Tests

Visual field impairment (VFI) will be assessed using a Humphrey 750i visual field analyzer (Carl Zeiss) and the central 30-2 SITA fast strategy protocol. Only tests that met the criteria (low [<20%], false positive, false negative, and fixation loss parameters) were evaluated [84].

Optical coherence tomography (OCT) images, assessing the central retinal thickness (CRT), macular ganglion cell–inner plexiform layer (GCIPL) thickness, and retinal nerve fiber layer (RNFL), will be performed using Cirrus HD-OCT 5000 with AngioPlex OCT-A (Carl Zeiss Meditec V.10.0) and cube scans. Only images with a signal strength of >6 with no movement artifact will be included in the study.

Two consecutive optical coherence tomography angiography (OCTA) images will be obtained by a single skilled examiner. A foveal centered scan area of a 3×3 mm^2^ pattern will be used, and all scans will be analyzed using en face OCTA images generated automatically with the optical coherence tomography microangiography (OMAG) algorithm in Angioplex software (ZEISS). The foveal avascular zone (FAZ) area will be manually outlined and expressed as square millimeters.

The vascular density (VD) will be measured automatically by the software, which will quantify the VD of a local region of tissue according to the ETDRS subfields. We will analyze the VD of each ETDRS sector, the inner ring, and the full area in 3×3 mm^2^ rings and exclude OCTA images with a signal strength of <7 [12].

The axial length will be calculated using an IOLMaster 500 optical biometer (Carl Zeiss Meditec) to be used as a correcting variable in the analysis, if necessary.

##### Questionnaires and Diaries

The State-Trait Anxiety Inventory (STAI), the Epworth Sleepiness Scale (ESS), the Pittsburgh Sleep Quality Index (PSQI) questionnaires and a sleep diary will be used to ask patients about their level of sleep quality, perceived stress, and anxiety.

##### Sleep Diary

During the 2-week period before trial commencement and the 4-week period of the intervention, patients will fill a daily self-recorded sleep diary at home. The sleep diary will record parameters such as bedtime, wake time, sleep onset, and sleep offset time.

##### State-Trait Anxiety Inventory

STAI is designed to assess the degree of state and trait anxiety in research participants (in this study, patients with moderate and severe RP) and takes about 10 minutes to complete. It is a reliable, valid, and responsive questionnaire consisting of 20 items for assessing state anxiety and 20 for trait anxiety. Higher scores (maximum score of 40) are positively correlated with higher levels of anxiety [85]. To evaluate the magnitude of any treatment effect, the minimum clinically important difference (MCID) will be regarded as a 30% reduction from baseline [86]. This magnitude represents a “small” effect in the behavioral sciences.

##### Epworth Sleepiness Scale and Pittsburgh Sleep Quality Index

The ESS and the PSQI are both sleep quality questionnaires. The ESS is widely used in the field of sleep medicine as a subjective measure of a patient’s sleepiness. The overall score ranges from 0 to 24. The scale estimates whether the patient is experiencing excessive sleepiness that possibly requires medical attention [87]. The PSQI is a self-reporting questionnaire that assesses sleep quality over a 1-month time interval. The global PSQI score is calculated by adding the 7 component scores, providing an overall score ranging from 0 to 21, where lower scores denote a healthier sleep quality [88].

As determination of the MCID in the sleep quality questionnaires remains controversial, with no consensus on methodology, we will estimate the minimum clinically important improvement in the ESS and PSQI to lie between −2 and −3 for this trial [88-92].

##### Laboratory Assessment

All patients will undergo a salivary and hair draw to confirm eligibility. Sample collection will be carried out between 9:00 A.M. and noon to minimize potential errors associated with diurnal variations in neuroendocrine parameters. Samples will be collected in the same room, and temperature and humidity will be recorded. After collection, samples will be frozen at –20 °C until analysis. When analyzing results, in female subjects, menstrual cycle phases will be considered.

##### Salivary α-Amylase, Cortisol, and Melatonin levels

Salivary samples will be collected by patients using the passive secretion drooling method. Two samples of saliva from each patient will be collected in 24 hours. At least 1 mL is required for analysis. The putative salivary indicators will be assayed with enzyme-linked immunosorbent assay (ELISA): cortisol (DRG Salivary Cortisol ELISA, DRG Instruments GmbH) and melatonin (DRG Instruments GmbH). Salivary α-amylase (sAA) will be determined with an α-amylase kit (Salimetrics) [93].

##### Cortisol Levels in Hair Samples

Three single 3-cm-long locks of hair will be collected from each patient to measure hair cortisol levels in a 3-month period (assuming an average growth rate of 1 cm per month). After collection, each hair sample will be wrapped in aluminum foil to protect it from light and humidity until analysis. The cortisol level in each sample will be measured using the Cortisol Hair ELISA kit (Alpco Diagnostics) with phosphate-buffered saline (PBS) at pH 8.0.

#### Randomization

All patients who provide written informed consent for participation and meet the eligibility criteria will be randomized and assigned using the online SAS Proc Plan permuted-block randomization with variable block sizes and assignments in sequentially numbered codes provided by the central randomization service on its website [94]. The randomization code will not be released until the patient has been recruited into the trial after baseline measurements have been completed.

An administrative assistant not otherwise involved in the study will provide the next treatment code in the sequence and will record the assignment in a confidential log.

#### Blinding

In our double-blinded trial, the principal investigator and statistician will be blinded to treatment allocation. Staff responsible for recruitment and grading severity will not be allowed to receive information about the group allocation. Raters will not be present during the lab analysis. Randomization will be conducted by the central randomization service to keep the principal investigator and statistician blind to the data management and study condition.

In the event of a SUSAR (suspected unexpected serious adverse reaction), if requested by the Data Safety Monitoring Committee (DSMC), or if a medical emergency occurs where knowledge of the blinded treatment is necessary, unblinding will be performed independently of the group allocation. The reporting of all SUSARs needs to be expedited.

### Intervention

All patients will be provided with a leaflet with clear instructions at the baseline visit, and they will be asked to follow the guidelines throughout the 4-week follow-up period.

#### Optical Filters for Blocking SWL

Patients will be provided with SWL-restricting goggles to wear during the daytime, in indoor bright-light conditions, and when using LED electronic devices in order to prevent the suppression of melatonin for at least 30 days. Optical filters will allow selective exclusion of less than 480-nm-wide wavelengths, while maintaining relatively good contrast sensitivity and a visual light transmittance of 70%-75% [95].

#### Oral Melatonin (6 mg)

Patients will be asked to intake a 6 mg melatonin dose orally once a day for 30 days in the evening (22:00 P.M. with a margin of 30 minutes before and after) to activate its sleep-promoting effects approximately 2 hours after intake, avoiding fatigue and sleepiness during the daytime [96-99].

Although the chosen dose is safe to obtain the desired effect, patients will be prompted to report any possible side effects, if present, during the intervention period [79,81,82,100]. If they experience unbearable side effects, they will have to discontinue the OM intervention. If they wish, they can participate in the SWL-blocking intervention and will be evaluated over the 4-week follow-up period.

#### SWL Blocking + Oral Melatonin (6 mg)

Participants in this combination arm will receive both interventions—filter goggles and OM—at the same time under the same conditions as the SWL and OM groups and for the same 30-day period.

#### No Intervention

The purpose of this arm is to serve as the procedural control, so patients in this group will be asked to fill their sleep diaries at home only for the first 2-week period after trial commencement. They will be chosen as comparator to avoid performance bias and other biases related to loss of binding in the trial.

#### Adherence

Adherence monitoring will be performed to find new strategies to avoid attrition during the study and to look for unexpected problems that may prevent patients from attending the sessions. Patients will receive weekly phone calls to confirm appointments and obtain educational instructions about the study protocols to reassure them of the importance of adherence. Upon discharge, patients will be provided with instructions to contact their physician for unacceptable levels of sleep disability or perceived stress to receive an outpatient prescription of antianxiety medications and lifestyle techniques. Additionally, if a patient deviates from the intervention protocol assigned to them, they will not be considered a study failure. Instead, we will continue to follow the patient through study completion and continue to track patient-reported outcomes.

### Data Collection and Outcome Measures

#### Baseline Assessments and Frequency of Follow-Up Assessments

A full list of assessments obtained at each time point is summarized in Table 3.

We will obtain baseline assessments during each patient’s screening visit. These measures will include, but will be not limited to, demographics, comorbidities, subjective sleep quality and perceived stress rating, ophthalmic and laboratory outcomes, and patient-reported outcomes specific to the planned intervention.

Initial follow-up assessments will be obtained at 1, 2, 3, and 4 weeks after the start of the intervention. Patients will also be asked to return for postintervention follow-up visits 6 months after the intervention. These visits will be scheduled at the beginning of the study. Patients unable to attend any in-person visit will be asked to at least provide all subjective patient-reported outcomes over the phone or via an electronic survey for that visit in order to facilitate retention and optimal follow-up.

Sleep quality level, laboratory outcomes, ophthalmic clinical outcomes, questionnaire patient-reported outcomes, and potential complications will be gathered during these visits.

**Table 3:** Assessments and follow-up.

Clinical and laboratory event	V_–1_^a^ (–4 weeks)	V_0_ (0 weeks)	V_1_ (1 weeks)	V_2_ (2 weeks)	V_3_ (3 weeks)	V_4_ (4 weeks)	V_F_ (6 months)
Screening	✓^b^	—^c^	—	—	—	—	—
Clinical	—	✓	—	—	—	✓	✓
Physical examination	—	✓	—	—	—	✓	✓
Salivary sample collection	—	✓	✓	✓	✓	✓	—
Hair sample collection	—	✓	—	—	—	✓	—
STAI^d^ questionnaire	—	✓	—	—	—	✓	—
ESS^e^ and PSQI^f^ questionnaires	—	✓	—	—	—	✓	—
Inclusion criteria	✓	—	—	—	—	—	—

^a^V: visit; V_–1_, screening visit, when inclusion and exclusion criteria will be checked; V_0_, visit to assess baseline characteristics; V_1_-V_4_, visits during the intervention period (every week); and V_F_, final visit after 6-month follow-up.

^b^✓: applicable.

^c^Not applicable.

^d^STAI: State-Trait Anxiety Inventory.

^e^ESS: Epworth Sleepiness Scale.

^f^PSQI: Pittsburgh Sleep Quality Index.

#### Primary Outcome Measures

The primary outcomes are the differences in changes from baseline to postintervention in hormone release (sAA, cortisol, and melatonin levels in salivary and hair samples) and sleep quality, as measured by the ESS and the PSQI.

#### Intra-Assay Variations

To evaluate the reproducibility of each salivary and hair biomarker, the within-subject standard deviation (Sw) will be calculated by obtaining the square root of the sum of the within-subject variance and the error variance estimated in a linear random-effects model. The mean intra-assay coefficients (ICCs) will be determined for each biomarker and interpreted as follows: 0 (SD 0.2), poor agreement; 0.3 (SD 0.4), fair agreement; 0.5 (SD 0.6), moderate agreement; 0.7 (SD 0.8), strong agreement; and >0.8, almost perfect agreement.

#### Secondary Outcome Measures

Secondary outcome measures include any clinical macular changes or retinal function measured with the ETDRS scale, CSV-1000 chart, and ancillary tests, such as the VFI, OCT, and OCTA. In addition, several patient-reported variables, such as self-recorded sleep diaries, perceived stress, and functional status will be recorded.

#### Covariates and Confounders

Several variables will be measured as potential covariates, including preintervention sleep quality scores, concomitant procedures, medical comorbidities, alcohol use, antianxiety medications or other nonprescribed medications used to help with insomnia and anxiety relief, tobacco use, and nonsteroidal anti-inflammatory drug (NSAID) and others anti-inflammatory drug treatment. In addition, basic demographic information will be obtained.

### Ethical Considerations

The protocol was reviewed by the local Institutional Review Board of the East Health Area of Valladolid (CASVE 21-520) and will be registered with ClinicalTrials when a start date is decided.

Informed consent will be obtained from each patient in the preintervention period, which must be signed, thumb-imprinted, or recorded by the patient and collected by a coached research assistant, when necessary.

Privacy and confidentiality will be guaranteed for each patient, and the study will be conducted in accordance with the principles of the Declaration of Helsinki.

### Data Management, Analysis, and Additional Information

#### Data Management

In the clinical trial, data handling will be managed by a third-party agency. The investigators, patients, and clinical staff rating or applying therapies will not have access to information until the data analysis is completed by predetermined statisticians. They will also not be aware of the allocation of patients in the trial. Data entry into the virtual system will also be managed by the third-party agency.

Patients’ information will be encrypted in a virtual database, secured, and password-protected. Only the data monitoring committee (DCM) will have access during the trial. Data coding will begin at randomization; SAS will generate an alphanumeric code for each block. An ID number will be assigned to each patient. If a major adverse effect occurs, it will be identified by the ID number.

The software will record any changes made by any person involved in the functioning of the program. The user ID will identify people who make any changes to the system, and this information will be used for the purpose of auditing the trial. During a regulatory audit, the auditors can verify any changes that occur in the system and data. All data will be regularly backed up to a password-secured database.

#### Data Monitoring

The DCM will comprise a data manager, a database programmer/designer, a medical coder, a clinical data coordinator, a quality control associate, and a data entry associate. A member of the DCM can have 1 or more duties. The DCM will be established according to the Spanish Agency of Medicines and Medical Devices (AEMPS) rules. According to Royal Decree 1090/2015, of December 4, the AEMPS is the regulatory authority responsible for clinical trial oversight, approval, and inspections in Spain and is attached to the Ministry of Health. The Institutional Ethics Committee will review and approve the clinical trial applications prior to the AEMPS initiating its review and approval process.

Data monitoring will take place in a pre-established room, with easy access to data, inside the institution but not in the same room where the trial is performed.

#### Interim Analysis

The clinical trial will have 4 major evaluation time points of interventions, that is, every week after V_0_ and 4 weeks and 6 months postintervention. In each visit for application of the intervention, the patient’s personal diary will be reviewed by the clinical staff. They have the responsibility to notify the DMC about eventual minor or major adverse effects.

Adverse events will be recorded from the start of stimulation through the end of the study. All adverse events regardless of attribution to OM and any unexpected adverse event due to the optical filters will be recorded using standard adverse event forms. Adverse events will be measured in several areas: seriousness, severity, length of duration, and any causal relationship, if any, with the intervention. Serious adverse events will be defined as those that are life threatening/disabling, require hospitalization, or result in death.

Patients who develop any adverse effect will be followed up. The DCM will have unblinded access to all patient data and will discuss the adverse events at a meeting. The decision of allowing a patient to withdraw from the study will be made by a majority consent of 50% or more.

### Statistical Analysis

#### Sample Size Calculation

A sample size estimation of 48 patients (n=12, 25%, per group) was calculated for a target power of 80% and considering an α (probability of rejecting a true null hypothesis) of 5% and an actual power of 80.02%. Cohen d was 1.2 [101]. Due to the possible overestimation of the effect size in pilot studies, an effect size of 0.6 (medium-high) was chosen for this study. We calculated a total sample size of 60 participants (a total of 15, 25%, patients per group), adding approximately 20% (3 more patients per group than before) to manage possible dropouts [102-105].

#### Statistics

A linear mixed effect regression model will be applied to compare intervention arms against the control at baseline and stimulated measures for the HPA axis or the retinal–SCN–pineal axis comprising cortisol, melatonin, and sAA [106]. Baseline characteristics of patients from each group will be compared applying independent *t* tests, 2-way repeated-measures ANOVA, and the Fisher exact test. To grade the RP severity (by quantifying clinical features, the ETDRS score, and visual field deviation) and the primary outcomes, we will consider both to be continuous variables and apply a multiple linear regression model. For secondary outcomes, normally distributed variables will be statistically assessed via parametric tests, such as the *t* test or ANOVA, while nonnormal distributed variables will be assessed with nonparametric tests, such as the chi-square test or the Fisher exact test and the Mann–Whitney U test. A chi-square or Fisher test will be performed to compare STAI, PSQI, and ESS scores among groups.

Statistical associations between covariates and outcomes will be evaluated using the appropriate statistical methodology, as previously described. Multivariable linear regression models will be appropriately fitted to continuous data based on the distributions of those data. Multiple logistic regression models will be fitted for all dichotomous outcome variables. Appropriate variable selection methods and model fit statistics will be used to determine the best-fitting model to determine the effects of treatment after adjusting for significant effects of covariates.

Finally, any protocol amendment will be first submitted to the Institutional Review Board for approval. If the amendment changes anything about what is reported

on ClinicalTrials, then the study’s registration on ClinicalTrials will be updated accordingly.

#### Modification/Discontinuation

If patients experience unbearable side effects related to melatonin, they must leave their intervention group. If they wish, they can continue participating in the study as part of the SWL group being evaluated over the 4-week follow-up period. To reflect these changes at the end of the study, an intention-to-treat analysis will be conducted.

#### Missing Data

Dropouts and missing facts are known as missing data. A dropout rate of 5%-10% is expected based on recent OM clinical trials [99,107]. An intention-to-treat analysis, regardless of the treatment administered, will be carried out for every primary and secondary endpoint. A preprotocol analysis will also be conducted with those who complete the trial, since secondary outcomes could be more affected by uncensored withdrawals. Both analyses must be favorable for the intervention to be considered better than standard. To deal with missing data, imputation techniques will be applied.

### Dissemination

Once the study enters the data reporting phase, the data sets used and analyzed during the study will be available from the corresponding author upon reasonable request. The protocol and results will be released on the ClinicalTrials website, as required upon study completion. Final study results will be published in a peer-reviewed, PubMed-indexed journal to reach health care professionals. The authors will also seek to present the study findings at relevant ophthalmic subspecialty society meetings. The Federación de Asociaciones de Retinosis Pigmentosa de España (FARPE) website will describe this project. Final findings and results will be published on this website for the general public, as well as disseminated through the FARPE social networking channels.

## Results

This work is a clinical trial protocol that has not yet been launched. The bibliographic research for information for its justification began in 2020, and this working group is currently seeking start-up funding. As soon as we have the necessary means, we will proceed with the registration and organization prior to the preliminary phase.

## Discussion

### Summary of Findings

The aim of the implementation of this clinical trial is to find an innovative strategy that allows a holistic approach for patients with RP, focusing on slowing down the progression of the disease, if possible, and improving their quality of life, especially their mood problems as well as their sleep-wake rhythm disorders.

The use of optical filters blocking SWL in the daylight was chosen as an intervention because of its nature as a noninvasive strategy as well as its protective potential against retinal neurodegeneration and melatonin suppression, both of which are relevant in these patients.

One of the main known sources of blue light is sunlight, but the widespread use of high-brightness lights with LED technology had made an increasing contribution in the past decade. A significant part of the LED emission spectrum peaks at 450 nm, within the wavelength range of blue light. Thus, with the increasing popularity of blue-rich LED-blacklight display devices, such as smartphones and tablets, computer screens, and other commonly used devices, our eyes are exposed to more blue light than they were in the past [28,36,75,108].

In addition, shorter-wavelength blue light has been reported to play a pivotal role in glare disability, especially in patients with retinal dystrophies [38,76]. Photophobia occurs because RPE cells appear to be defective with regard to absorbing light and because a lack of photoreceptors makes the adaptation to different lighting levels difficult [38,76]. Several studies have shown that patients with RP present higher values of intraocular scattering and, therefore, a decreased visual discrimination capacity in comparison to healthy people [77,109-111].

However, although excessive blue light is theoretically harmful, some studies have shown that longer blue wavelengths of the visible spectrum (465-495 nm) are essential for the circadian rhythm, pupillary reaction, color discrimination, and night vision [7,23,24,40-43]. Therefore, optical SWL filters may offer interesting advantages in having a positive effect on the visual discrimination capacity and decreasing glare disability in patients with RP. Finally, they could also help in circadian rhythm regulation and modulate the spectral transmittance of SWL, protecting injured eyes from potential additional photochemical damage.

We have selected melatonin as an intervention because of its close relationship with and involvement in the regulation of circadian cycles and its antioxidant and anti-inflammatory potential.

Melatonin’s functions are either receptor dependent or receptor independent. The former includes circadian rhythm control and sleep regulation. There is a considerable body of evidence of melatonin regulating sleep in various sleep disorders [7,62-66]. In fact, melatonin appears as one of the most effective drugs for treating circadian rhythm sleep-wake disorders [67]. Aging, the presence of certain diseases (eg, primary degeneration of the autonomous nervous system and diabetic neuropathy, some types of neoplasms, Alzheimer disease), and certain drugs (eg, β-blockers, clonidine, naloxone, and NSAIDs) abolish the nocturnal production of melatonin and are associated with impaired sleep [7,61,79,81].

Melatonin acts via its own receptors (MT1, MT2) present at the SCN, which are members of the G protein–linked receptor family [61]. The phase-shifting effects of melatonin are essentially opposite to those of light. In humans, its sleep-promoting effects become significant about 2 hours after intake, similar to the physiological sequence at night. However, administration of melatonin during the daytime (when its endogenous levels are minimal) results in induction of fatigue and sleepiness in humans [79,81].

Thus, melatonin administration appears to be one of the most promising approaches, with long-term benefits, for circadian rhythm sleep disorders because it simultaneously treats sleep and wake state problems and, in addition, synchronizes other bodily rhythms so as to maintain the body’s internal temporal order and prevent internal desynchronization [67,79,81].

Although the receptor-independent functions of melatonin include the detoxification of ROS and other reactive molecules, the antioxidant action of melatonin can also be expressed by increasing the efficacy in preventing oxidative DNA damage [7,51,58,60,61].

In addition to its pleiotropic actions, melatonin possesses an advantage over other possible candidates due to its amphiphilic character, which enables it to enter any fluid, cells, or subcellular structures, including mitochondria. Conversely, melatonin exhibits poor pharmacokinetic properties (eg, limited oral bioavailability and short plasma half-life) and low subtype receptor selectivity [79,81]. The effective doses reported are between 0.5 and 10 mg without a clear dose-response relationship (Multimedia Appendix 1). This mainly reflects the large interindividual variability in melatonin bioavailability, which is well known in the published literature and is attributed to differences in first-pass metabolism (Multimedia Appendix 1).

The acute toxicity of melatonin, as seen in both animal and human studies, is extremely low [82]. Melatonin may cause minor adverse effects, such as headache, insomnia, rash, diarrhea, and nightmares [82]. In animals, a lethal dose for 50% of the subjects (LD50) could not be established; even 800 mg/kg body weight (high dose) was not lethal [100]. Studies on human subjects given varying doses of melatonin (1-6.6 g/day) for 30-45 days and followed with an elaborate battery of biochemical tests to detect potential toxicity have concluded that in addition to drowsiness, all findings were normal at the end of the test period [79,81,82].

In the same line as the previous Spanish RCT (EudraCT no: 2012-002436-82) with OM and patients with RP, we have considered that 6 mg of OM for 30-40 days will be a safe dose to reach the desired effects without causing side effects in our target population. In general, a dose between 0.2 and 10 mg has been considered a safe starting dose in most of the previous studies with OM [67,79,81,82].

Accumulating evidence indicates that melatonin has a protective effect against various ocular disorders, such as uveitis, diabetic retinopathy, glaucoma, and even optic neuritis, through different mechanisms of action [60,61,73,112-115]. The multifaceted role of melatonin in neuroprotection might imply that melatonin may possess the capacity to attenuate the harmful effects that occur in degenerative retinal diseases, such as RP. Daily administration of melatonin has been shown to delay the photoreceptor apoptotic loss in RP mice [53,116]. This evidence supports that a clinical trial using melatonin to at least delay cell loss in RP in humans might be beneficial. In this context, a Spanish RCT (EudraCT no: 2012-002436-82) with 6 mg of OM and patients with RP is ongoing. Thus, therapeutic treatments of eye diseases using melatonin alone or in combination with other strategies deserve serious investigation.

The possibility of synergy between the 2 strategies stems from the common point between them—their involvement in circadian rhythms. Glucocorticoid hormones, particularly cortisol, participate in the body’s homeostasis and stress responses and follow a circadian rhythm, exhibiting a predictable peak in the morning, with a typically sharp elevation 30 minutes to an hour after awakening [117-119], although one that is apparently more complex than the melatonin rhythm.

The sAA enzyme has been used as a marker for the sympathetic nervous system response [118,120,121] and, like cortisol, has been shown to respond to psychosocial stress [118]. As with melatonin and cortisol, sAA production exhibits a regular circadian pattern [118]. Under normal, steady-state conditions, high levels of cortisol tend to be associated with low levels of sAA and vice versa.

Synthesis of melatonin is affected by SWL, whereas cortisol production and sAA levels are modulated by both short- and long-wavelength light. Since light is a well-known stimulus for suppressing the synthesis of the hormone melatonin at night, it was considered important for this study to also understand how blocking SWL in bright-light conditions may prevent the suppression of melatonin, which could affect perceived stress, sleep quality, and the quality of life of patients with RP.

### Limitations

The limitations that may be encountered in the application of this clinical trial are likely to be related to the large phenotypic and genotypic variability found among patients with RP; the difficulties in recruiting patients who meet the eligibility criteria, especially those with enough visual acuity that allows them to use the filters; and the subjective nature of the results of the questionnaires. However, this could be overcome by a good baseline study that allows for proper analysis that considers possible confounding variables. However, a sleep-monitoring device could be used, such as second-generation multisensory sleep trackers, but their price would increase the cost of the study and there is still no validated device for this purpose, so for the time being, they have not been included in the study protocol [122].

### Conclusion

Preservation of vision in retinal degeneration is one of the priorities of the World Health Organization Vision 2020 program. Clinical trials of melatonin in the context of controlling RP, for example, warrant serious consideration. Furthermore, positive results of such studies would not only benefit patients with various ocular disorders but also be particularly helpful in countries where the treatments are expensive and not easily accessible.

This protocol for a double-blind RCT aims to shed light on the benefits that OM combined with SWL-blocking filters might have for patients with moderate or severe RP in terms of disease progression and quality of life. The goal is that this protocol be replicated for future studies, applied to additional inherited retinal dystrophies, and potentially incorporated into clinical practice. Should this study demonstrate the superiority of the combined intervention versus no intervention, its findings may also help reduce the impact of sleeping disorders and negative psychological disorders on the quality of life of patients with RP and help them recover their self-autonomy.

## Appendix

Multimedia Appendix 1 Supplementary table with all existing randomized controlled trials registered at ClinicalTrials.gov to date using oral melatonin.

## Abbreviations

AEMPS: Spanish Agency of Medicines and Medical Devices
DCM: data monitoring committee
ELISA: enzyme-linked immunosorbent assay
ESS: Epworth Sleepiness Scale
ETC: electron transport chain
ETDRS: Early Treatment Diabetic Retinopathy Study
FARPE: Federación de Asociaciones de Retinosis Pigmentosa de España
HPA: hypothalamic–pituitary–adrenal
IOBA: Institute of Applied Ophthalmobiology
ipGC: intrinsically photosensitive retinal ganglion cell
LED: light-emitting diode
MCID: minimum clinically important difference
NSAID: nonsteroidal anti-inflammatory drug
OCT: optical coherence tomography
OCTA: optical coherence tomography angiography
OM: oral melatonin
PSQI: Pittsburgh Sleep Quality Index
RCT: randomized controlled trial
ReCYL: Retina Castilla y León
ROS: reactive oxygen species
RP: retinitis pigmentosa
RPE: retinal pigment epithelium
sAA: salivary α-amylase
SCN: suprachiasmatic nucleus
STAI: State-Trait Anxiety Inventory
SUSAR: suspected unexpected serious adverse reaction
SWL: short-wavelength light
VD: vascular density
VFI: visual field impairment

## Data Availability

All data sets generated and analyzed during this study are included in this published paper and Multimedia Appendix 1. Once the study enters the data reporting phase, the data sets will be available from the corresponding author upon reasonable request.
