# Supplementary material for: Short-Wavelength Light–Blocking Filters and Oral Melatonin Administration in Patients With Retinitis Pigmentosa: Protocol for a Randomized Controlled Trial
Source: JMIR Res Protoc. 2023 Nov 16;12:e49196. doi: 10.2196/49196 (PMC10690531; doi:10.2196/49196)
Supplement: Multimedia Appendix 1 [file resprot_v12i1e49196_app1.pdf]

CLINICAL TRIALS

Number	Name	Condition	Age	year	Location	Purpose	Dose	Posology	duration	vs
NCT02889432	Effects of Oral Melatonin on Neurosensory Recovery Following Facial Osteotomies	Dentofacial Deformities	18-40 y	2016	Hong kong	treatment	10 mg	30 min before bedtime	3 wk	Placebo
NCT03732963	Oral Melatonin as Premedication During MAC for Patients Undergoing Loco-regional Chronic Subdural Hematoma Evacuation	Loco-regional Chronic Subdural Hematoma Evacuation	50-65 y	2018	Egypt	Supportive care	10 mg	preoperatively		Placebo
NCT03000777	Oral Melatonin Plus Zinc Supplementation in Chronic Fatigue Syndrome/Myalgic Encephalomyelitis (CFS/ME)	Fatigue Syndrome, Chronic|Myalgic Encephalomyelitis	18-65y	2016	Spain	treatment	10 mg	1 h before bedtime (Melatonin+zinc)	16 wk	Placebo
NCT00470821	Oral Melatonin in Critically Ill High-risk Patients	Critically Ill Patients|Mechanically Ventilated Patients	18-85 y	2007	Italy	treatment	3 mg	at 8 pm and 12 pm		placebo
NCT03764020	Trial of Oral Melatonin in Elevated Blood Pressure	Elevated Blood Pressure	30-60 y	2018		treatment		1 h before bedtime	3 wk	placebo
NCT04235673	Oral Melatonin as Neuroprotectant in Preterm Infants	Preterm infants	25-30 wk	2020	Italy	Supportive care	3 mg/kg	c 24h	15 dy	Placebo
NCT02430298	Topical/Oral Melatonin for Preventing Concurrent Radiochemotherapy Induced Oral Mucositis/Xerostomia Cancer Patients	Head and Neck Cancer	18-60 y	2015	Thailand	Prevention	20 mg	2 min before radiation and 15 min at 21:00	7 wk	Placebo
NCT01922999	Different Doses of Oral Melatonin Supplements in Chronic Kidney Disease (CKD)-Associated Sleep Disorders	End Stage Renal Disease	18-85y	2013	US	treatment	3 mg	c 24h	60 dy	placebo
NCT03631901	Comparison Between Melatonin and Diazepam for Prevention of Recurrent Simple Febrile Seizures	Febrile Seizures	6 mt-5 y	2018	Egypt	Prevention	0,3 mg/kg	c 8h	during febrile illness	diazepam
NCT02332928	Melatonin Supplementation for Cancer-related Fatigue in Patients Receiving Radiotherapy	Breast Cancer - Female	18y	2015	US	Supportive care	20 mg	c 24 at night before their first RT	2 wk	placebo
NCT03826563	Melatonin Effects on Sleep and Circadian Rhythm in Youth and Young Adults With At-risk Symptoms	Psychosis|Sleep	11-30 y	2019	US	treatment	5 mg 1 wk + 2,5-10 mg 1 wk	c 24h nightly	2 wk	Placebo
NCT02591238	Melatonin in Smoke-induced Vascular Injury	Smoking|Vascular System Injuries	25-39 y	2015	China	treatment	3 mg	c 24h	2 wk	Placebo
NCT01805089	Melatonin Versus Placebo in Breast Cancer	Breast Cancer	18 y	2013	US	treatment	3 mg	at 9 pm	4 mt	placebo
NCT02359448	Melatonin for Nocturia in Parkinson's Disease	Parkinson's Disease|Nocturia	18 y	2015	UK	treatment	2 mg	c 24h	6 wk	not controlled
NCT02639741	Preoperative Melatonin or Vitamin C Administration on Postoperative Analgesia	Postoperative Pain	18-65 y	2015	turkey	treatment	6 mg	preoperatively		vitamin c and placebo
NCT02265822	Melatonin Premedication in Children Undergoing Surgery	Anaesthesia	5-14y	2014		treatment	0,5 mg/kg (max 20 mg)	40 min before induction		midazolam
NCT03995004	The Effect of Melatonin on Postoperative Pain Reduction After Orthognathic Surgery	Effect of Drugs	18-60y	2019		treatment	10 mg	before the surgery at night	4 dy	Dexamethasone
NCT01355523	The Effect of Melatonin on Depression, Anxiety, Cognitive Function and Sleep Disturbances in Breast Cancer Patients	Breast Cancer|Depression	30-75 y	2011	Denmark	treatment	6 mg	1h before bedtime	12 wk	Placebo
NCT01114360	Melatonin and Nighttime Blood Pressure in African Americans-8 mg Study	Hypertension	18-64 y	2010	US	treatment	8 mg	c 24h time-relese	4 wk	Placebo
NCT01114373	Melatonin and Nighttime Blood Pressure in African Americans--24 mg Study	Hypertension	18-64 y	2010	US	treatment	24 mg	c 24h time-relese	4 wk	Placebo
NCT04005222	Effects of Selenium and Melatonin on Ocular Ischemic Syndrome	Anterior Segment Ischemia (Diagnosis)	61-75 y	2019		Prevention	0,5 mg/kg	c 12h	4 wk	Placebo
NCT01161108	Trial of Melatonin to Improve Sleep in Children With Epilepsy and Neurodevelopmental Disabilities	Epilepsy|Insomnia|Developmental Disability	5-17 y	2010	Canada	treatment	3 (<20 kg) /6 mg fast relese	1h before bedtime	7 wk	3/6 mg timed relese
NCT04278677	Sleep Quality and Pain Medication Use Following Arthroscopic Rotator Cuff Repair	Sleep Disturbance	18 y	2020	US	treatment	5 mg	c 24h	6 wk	
NCT02195661	The Role of Melatonin in the Effective Attainment of Sleep Electroencephalograms (EEG) in Children	Epilepsy|Electroencephalogram|Children|Sedation	6 mt-13 y	2014	South Africa	Diagnostic	3 mg (<15mg) / 6mg	1h before bedtime		
NCT04137627	Melatonin Effect in Combination With Neoadjuvant Chemotherapy to Clinical Response in Locally Advanced Oral Squamous Cell Carcinoma	Oral Squamous Cell Carcinoma|Neoadjuvant Chemotherapy	child, adult	2019	indonesia	Supportive care	20 mg	in addition to neoadjuvant chemotherapy		Placebo
NCT02631148	Effects of Melatonin Supplementation on Renal Physiology in a Habitual Sleep Restricted Population.	Obesity|Prediabetic State	18 y	2015	US	basic science	2 mg	1h before bedtime	6 wk	Placebo
NCT03368430	Melatonin as an Adjunctive Therapy for Chronic Periodontitis.	Periodontal Diseases	35-60 y	2017	Egypt	treatment	10 mg	1h before bedtime	2 mt	Placebo
NCT02099331	Cardiac Surgery and the Risk of Atrial Fibrillation: an Intervention Trial Evaluating Melatonin	Atrial Fibrillation	18 y	2014	US	Prevention	20 mg	prior to sleep	6 dy (2 prior 3 postop)	Placebo
NCT01724424	Melatonin in Healthy Volunteers	Sepsis	18-30 y	2012	UK	basic science	20-30-50-100 mg	single dose		20-30-50-100 mg
NCT01874847	PLAY GAME: Post-concussion Syndrome in Youth - Assessing the GABAergic Effects of Melatonin	Post-concussion Syndrome	8-19 y	2013	Canada	treatment	3 mg- 10 mg	once at night	28 dy	Placebo
NCT02319265	Dose Assessment of Melatonin in Sepsis Trial	Sepsis	16 y	2014	UK	treatment	50 mg -100 mg	single dose		Placebo
NCT03438526	The Basel BOMP-AID Randomized Trial	Hypoactive Delirium	55 y	2018	Switzerland	treatment	4mg	at 8 p.m.	during hypoactive delirium	Placebo
NCT04531748	Selective Estrogen Modulation and Melatonin in Early COVID-19	Covid19	18 y	2020		treatment	100 mg	10 mg in the morning 60 mg in the evening	10 mg 2 Sya 60 mg 11 days	Toremifeno y placebo
NCT03968939	Total Joint Arthroplasty and Sleep	Primary Total Hip Arthroplasty|Primary Total Knee Arthroplasty	18 y	2019	US	treatment	3 mg			with diphenhydramine y placebo
NCT02845778	Pharmacokinetics of Melatonin Niosomes Oral Gel in Healthy Volunteers	Pharmacokinetics of Melatonin	18-30 y	2016	Thailand	basic science	2,5-5-10 mg			AUC
NCT02630004	Melatonin Oral Gel for Oral Mucositis in Patients With Head and Neck Cancer Undergoing Chemoradiation	Oral Mucositis	18 y	2015	Spain	Prevention	3% oral gel		3 days before RT and 4 wks after	Placebo
NCT03788733	The Efficacy of Melatonin in the Burning Mouth Syndrome	Sleep Disorders, Circadian Rhythm	child, adult	2018	Spain	treatment	3mg	once at night	8 wks	Placebo
NCT03833570	Melatonin for Prevention of Radiation Induced Oral Mucositis	Oral Mucositis (Ulcerative) Due to Radiation	25 y	2019	Egypt	Prevention	10 mg	30 min before bedtime	6 wk	conventional therapy
NCT03951025	Study of the Bioavailability of a Food Supplement Rich in Melatonin Administered Sublingually and Orally (MELATONIN)	Biological Availability	18 y	2019	Spain	basic science	1 mg orally			1 mg sublingually
NCT02580734	Efficacy of Melatonin in Burning Mouth Syndrome (BMS)	Burning Mouth Syndrome	18-90 y	2015		treatment	12 mg	3 mg c6h	8 wks	Placebo
NCT04251845	Evaluation of Effect of Topical Melatonin in Treatment of Oral Leukoplakia	Oral Leukoplakia	18 y	2020	India	treatment	15 mg	once daily	6 wk	Placebo
NCT02107079	The Relative Bio-availability of Oral and Oromucosal Melatonin in Different Formulations in Healthy Human Volunteers.	Pharmacokinetics of Melatonin	18-35 y	2014	netherlands	basic science	1-2,5-0,5 mg inmediate release and oromucosal			
NCT03205033	Melatonin as a Circadian Clock Regulator, Neuromodulator and Myelo-protector in Adjuvant Breast Cancer Chemotherapy	Sleep Disorders, Circadian Rhythm|Depression|Genotoxicity|Pain	18-75 y	2017		treatment	20 mg	1h before bedtime	10 dy (7 before QT and 3 after)	Placebo
NCT04335968	Melatonin for Prevention of Postoperative Delirium After Lower Limb Fracture Surgery in Elderly Patients	Orthopedic Surgery	70 y	2020		Prevention	4 mg	once nightly	6 dy	Placebo
NCT03966950	Use of Melatonin for Preventing POCD in Transurethral Prostate Resection Under Spinal Anesthesia	Melatonin|Cognitive Dysfunction|Postoperative Complications|Prostate Hyperplasia	60 y	2019	brazil	Prevention	10 mg	once nightly	4 dy (1 pre, 1 cx, 2 post)	Placebo
NCT04353128	Efficacy of Melatonin in the Prophylaxis of Coronavirus Disease 2019 (COVID-19) Among Healthcare Workers.	Covid19|SARS-CoV 2|Coronavirus Infection	18-65 y	2020	Spain	Prevention	2mg prolonged release	once nightly	12wks	Placebo
NCT03150797	Melatonin for Adolescent Migraine Prevention Study	Migraine	10-17 y	2017	US	Prevention	3 mg	1h before bedtime	8 wks	Placebo

NCT04034771	The Effect of Melatonin Administration on Sedation Level as Adjuvant to Propofol	TBI (Traumatic Brain Injury)	18-65 y	2019	Egypt	Supportive care	10 mg	once at admision		propofol alone
NCT04788979	The Adjunctive Use of Melatonin Therapy in the Treatment of Obese Periodontitis Patients (Clinical and Immunological Study).	Periodontal Diseases	27-54 y	2021	Iraq	treatment	5 mg			
NCT03894683	Effect of Melatonin on Cardiovascular and Muscle Mass and Function in Patients With Heart Failure	Heart Failure With Reduced Ejection Fraction	30 y	2019	Iran	treatment	10 mg	at bedtime	6 mt	Placebo
NCT03258294	Effect of Melatonin on Sleep Disturbances in Patients With Parkinson's Disease	Parkinson's Disease	55 y	2017		treatment	2 mg	at bedtime	4 wk	Placebo
NCT04665453	Dexmedetomidine and Melatonin for Sleep Induction for EEG in Children	Epilepsy	1-20 y	2020	Slovenia	Diagnostic	0,1 mg/kg	before monitoring	once	Dexmedetomidine intranasal alone
NCT03043443	Melatonin Use for Sleep Problems in Alcohol Dependent Patients	Alcohol-Related Disorders	19 y	2017	Canada	treatment	5 mg	1h before bedtime	4 wk	Placebo
NCT03859934	Metabolic Effects of Melatonin Treatment	Type 2 Diabetes Mellitus	40-17 y	2019	Denmark	treatment	10 mg	1h before bedtime	3 mt	placebo
NCT03725267	Melatonin for Renal Protection in Patients Receiving Polymyxin B	Acute Kidney Injury receiving Polymyxin B	18 y	2018	brazil	treatment	30 mg	1h before bedtime	14 days	placebo
NCT03708341	Exogenous Melatonin in Intensive Care Unit Chronodisruption	Intensive Care Psychosis	18 y	2018	Lebanon	treatment	5 mg	at fixed time	during ICU stay	Placebo
NCT03204877	Acute Metabolic Effects of Melatonin Treatment	Glucose Metabolism Disorders	20-40 y	2017	Denmark	other	10 mg	every hours for 4 hours	study day	Placebo
NCT04574141	Kinetic of Melatonin Subsequent to the Consumption of Melatonin-rich Food Supplements	Melatonin Bioavailability	18-45 y	2020		basic science	1,9 prolongued relese tablet + 1 mg oral spray			1 mg oral spray + 1,9 prolongued relese tablet
NCT04785183	Antioxidant Effects of Melatonin in Preterm	Melatonin Deficiency	6h preterm	2021	Italy	treatment	melatonin drops			placebo
NCT02681887	Effect of Melatonin on Cardiometabolic Risk- FULL	Obesity|Prediabetic State	18 y	2016	US	basic science	2 mg	once before bedtime	12 wks	placebo
NCT04424875	The Effect of Melatonin Application Following Removal of Impacted Third Molar	Impacted Third Molar Tooth	18 y	2020	Egypt	Supportive care	3 mg	once on the surgery	once	placebo
NCT03879707	Improving Sleep Quality After Total Joint Arthroplasty (TJA)	Pain After TJA	18-99 y	2019	US	treatment	5 mg	c 24h (+magnesium)	14 wks	placebo
NCT01695070	Melatonin to Prevent Brain Injury in Unborn Growth Restricted Babies	Fetal Growth Retardation	18-45 y	2012	Australia	treatment	4 mg	prolonged relese c 12h	throughout pregnacies affected by CIR	placebo
NCT01858909	Efficacy of Melatonin in Patients With Severe Sepsis or Septic Shock	Severe Sepsis|Septic Shock	18 y	2013	Spain	treatment	30 mg	c 12h	28 dys	placebo
NCT02597231	Melatonin and Sleep in Preventing Delirium in the Hospital	Delirium	65-100 y	2015	US	Prevention	3 mg	once at 9 pm		placebo
NCT03656484	New Periodontitis Treatment Based on Hyaluronic Acid and Melatonin	Chronic Periodontitis	30-70 y	2018	Romania	treatment	0,18%	c 24h	30 dys	
NCT03540485	Safety and Efficacy of Melatonin in Patients With Multiple Progressive Primary Sclerosis	Sclerosis, Multiple	18-65 y	2018	Spain	treatment	300mg	once at 10-11 pm	24 mts	placebo
NCT02615340	Melatonin for Prevention of Delirium in Critically Ill Patients	Delirium	18 y	2015	Canada	Prevention	0,5-2 mg (enterally)	Once at 21-00	ICU discharge or 14 dys	0,5-2 mg - placebo
NCT02200172	The Preventative Role of Exogenous Melatonin Administration in Patients With Advanced Cancer Who Are at Risk of Delirium: a Feasibility Study	Cancer	18 y	2014	Canada	Prevention	3 mg (sublingual)	Once at 21.00	28 dys or discharge	placebo
NCT02108353	Circadian Phase Adjustment and Improvement of Metabolic Control in Night Shift Workers	Sleep Disorders, Circadian Rhythm	18 y	2017	Canada	Prevention	2 mg	1h before bedtime and 1h after the last meal	12 wks	placebo
NCT03498131	Melatonin in Patients With Multiple Sclerosis (MS).	Relapsing Remitting Multiple Sclerosis	18-65 y	2018	US	treatment	3-5 mg	once at 21.00	1 y	placebo (treated with DMT)
NCT04474483	Safety and Efficacy of Melatonin in Outpatients Infected With COVID-19	COVID-19	18 y	2020	US	treatment	10 mg	c 8 h	14 days	placebo
NCT03848533	Effect of Melatonin and Metformin on Glycemic Control Genotoxicity and Cytotoxicity Markers in Patients With Prediabetes	PreDiabetes	30-60 y	2019	Mexico	treatment	5 mg	lengthed release once a day in the night	90 dys	placebo and metformin 850 mg
NCT02663570	Effects of Melatonin in PCOS Women	Polycystic Ovary Syndrome	18-35 y	2016	Italy	treatment	2 mg	once a day	6 mt	not controlled
NCT04784754	Dose-Ranging Study to Assess the Safety and Efficacy of Melatonin in Outpatients Infected With COVID-19	COVID-19	18 y	2021	US	treatment	3 mg - 30 mg	c 8 h	14 dy	3-30 mg - Placebo
NCT01780883	Melatonin Dose-effect Relation in Childhood Autism	Childhood Autism	6-8 y	2013	France	treatment	2-4-10 mg	1h before bedtime	6 wks	2-4-10 mg - placebo
NCT02415309	Premedication With Melatonin in Lumbar Medial Branch Block Procedure	Anxiety	18-50 y	2015	US	treatment	2-10 mg	90 min before, during and 90 min post		2-10 mg - placebo
NCT00506064	Melatonin Postoperative Sleep Study in Breast Cancer Patients	Breast Cancer	40 y	2007	US	treatment	0,15 mg/kg (9 mg)	30 min before bedtime (10h)	1 wk	Placebo
NCT02798367	Melatonin 3mg and 5mg Compared to Cognitive Behavioral Therapy for Insomnia (CBT-I) in the Treatment of Insomnia	Insomnia Disorder	55 y	2016	brazil	treatment	3-5 mg	1h before bedtime	21 dy	3-5 mg - placebo
NCT00238108	Melatonin Supplements for Improving Sleep in Individuals With Hypertension	Sleep Disorders|Hypertension	35-65 y	2005	US	treatment	2,5 mg	once a day	3-4 wks	placebo
NCT01200641	Evaluation of Anxiolysis and Pain Associated With Retrobulbar Eye Block for Cataract Surgery : Melatonin Versus Gabapentin	Anxiety|Pain	35-85 y	2010	Iran	Prevention	6 mg	90 min before retrobulbar		gabapentin - placebo
NCT04229719	Effect of Topical Melatonin Application on Dental Implant Osseointegration and Marginal Bone Level	Alveolar Bone Loss	30-55 y	2020	Iraq	treatment	1,2 mg	topical application in osteotomy		no intervention
NCT00000171	Study of Melatonin: Sleep Problems in Alzheimer's Disease	Alzheimer Disease|Dyssomnias	55 y	1999	US	treatment	2,5 mg slow release - 10 mg inmediate	once a day	8 wks	other doses - placebo
NCT00927030	Melatonin for Sleep in Children With Autism	Autistic Disorder|Insomnia	4-10 y	2009	US	treatment	1-3-6-9 mg	30 min before bedtime	3 wks	bw doses and placebo
NCT04570254	Antioxidants as Adjuvant Therapy to Standard Therapy in Patients With COVID-19	Pneumonia, Viral|Covid19	child, adult	2020	Mexico	treatment	50 mg	c 24h	5 dys	with vit C, E, NAC, pentoxi
NCT02451293	The Effect of MElatonin on Depression, Anxiety, CIrcadian and Sleep Disturbances in Patients After Acute Myocardial Syndrome	Depression|Acute Coronary Syndrome	18 y	2015	Denmark	treatment	25 mg	1h before bedtime	12 wks	placebo
NCT01575678	The Effect of Melatonin on Nocturnal Enuresis	Nocturnal Enuresis	6-14 y	2012	Denmark	treatment	2-4 mg	once a day	4 wks	placebo
NCT02757079	Study of the Efficacy and Safety of NPC-15 for Sleep Disorders of Children With Neurodevelopmental Disorders	Neurodevelopmental Disorder	6-15 y	2016		treatment	1-2-4 mg	before bedtime	26 wks	placebo
NCT02454855	Impact of a Melatonin Supplementation on the Quality of Life in Elderly Metastatic Cancer Patients	Cancer	70 y	2015	France	Supportive care	2 mg prolongued release	1-2h before bedtime	3 mts	placebo
NCT01279876	Melatonin in Relapsing-Remitting Multiple Sclerosis Patients	Multiple Sclerosis, Relapsing-Remitting	20-45 y	2011	Iran	treatment	3 mg	1h before bedtime		placebo
NCT00544791	The Effect of Melatonin on Cognitive Function in Patients Diagnosed With Mild Cognitive Impairment	Mild Cognitive Impairment (MCI)	55-90 y	2007	Israel	treatment	5 mg	c 24 h	6 mt	placebo
NCT03785158	MIND After Surgery	Delirium Major Non-cardiac Surgery	65 y	2018	Canada	Supportive care	3 mg	7-9 pm once a day	8 dys	placebo
NCT03557229	Clinical Trial of Antioxidant Therapy in Patients With Septic Shock	Oxidative Stress|Septic Shock	18 y	2018	Mexico	treatment	5 mg	once daily	5 dys	vit C, vit E, NAC and placebo
NCT02732288	Exploratory Study of Melatonin Induced Sleep Regularization in Severe Brain Injury	Disorders of Consciousness	18-65 y	2016	US	basic science	3 mg	8 pm once a day	3 mts	healthy volunteers
NCT00675181	Effects of Melatonin and Oxygen Consumption and Choroidal Blood Flow	Vasospastic Syndrome	20-30 y	2008	Switzerland	basic science	5 mg	2 pm once		placebo
NCT02333149	Melatonin in Youth: N-of-1 Trials in a Stimulant-treated Attention Deficit Hyperactivity Disorder (ADHD) Population	Hyperactivity Disorder	6-17 y	2015	Canada	treatment	3-6 mg (>40 kg)	30 min-1h before bedtime	6 wks	placebo
NCT02768077	Effect of Melatonin on Sleep Disturbances in Patients With Parkinson's Disease	Parkinson's Disease	55 y	2016		treatment	2 mg prolongued release	before bedtime	4 wks	placebo

NCT02344316	Bringing Relief to Adolescents Naturally Using Melatonin for Migraine	Migraine in Adolescents	12-17 y	2015	US	treatment	3 mg	9 pm or 1h before bedtime	28 days	placebo
NCT02883790	Effects of SomnageÂ® in the Management on Sleep and Mood in Cancer Patients	Breast Cancer|Lung Cancer|Colon Cancer	18-75 y	2016	Italy	Supportive care	1 mg orally	once		with ZN and VRN
NCT04470297	Melatonin Agonist on Hospitalized Patients With Confirmed or Suspected COVID-19	Covid19|Lung Injury	18-80 y	2020	brazil	treatment	8 mg	once at bedtime	10 dys	placebo
NCT02282241	Melatonin for Delirium Prophylaxis	Delirium	65 y	2014	Canada	Prevention	1, 5 mg	in the evening	14 dys	placebo
NCT02963181	Effects of Melatonin to Reduce Nocturnal Hypertension in Patients With Neurogenic Orthostatic Hypotension	Hypotension, Orthostatic	18-80 y	2016	Canada	treatment	2-5 mg separate dosis	two separate dosis		yohimbine
NCT00513357	Melatonin Versus Placebo and the Effect on Appetite in Advanced Cancer Patients	Gastrointestinal and Lung Cancer	18 y	2007	US	treatment	20 mg	before bedtime	4 wks	placebo
NCT02642640	Interaction of Melatonin and MTNR1B Genotype on Glucose Control - Study 1	Glucose|Genes	21-55 y	2015	US	basic science	5 mg			placebo-mt mt-placebo
NCT00825591	Biological Clock Dysfunction in Optic Nerve Hypoplasia	Optic Nerve Hypoplasia	2-10 y	2009	US	Diagnostic	0,5 - 3 mg/m2	2 doses before beditme	6 wks	Placebo
NCT01557478	Melatonin as Adjuvant Therapy in Breast Cancer Patients	Stage II and III Breast Cancer	18-70 y	2012	Thailand	treatment	20 mg	once after 9 pm	24 mts	Placebo
